# Cryptococcosis in Hematopoietic Stem Cell Transplant Recipients: A Rare Presentation Warranting Recognition

**DOI:** 10.1155/2020/3713241

**Published:** 2020-08-19

**Authors:** Carolina Firacative, Silvia Katherine Carvajal, Patricia Escandón, Jairo Lizarazo

**Affiliations:** ^1^Studies in Translational Microbiology and Emerging Diseases (MICROS) Research Group, School of Medicine and Health Sciences, Universidad del Rosario, Bogota 111221, Colombia; ^2^Group of Microbiology, Instituto Nacional de Salud, Bogota 111321, Colombia; ^3^Internal Medicine Department, Hospital Universitario Erasmo Meoz, Universidad de Pamplona, Cucuta 540003, Colombia

## Abstract

Cryptococcosis, a life-threatening mycosis caused mainly by *Cryptococcus neoformans*, appears to be distinctly rare in hematopoietic stem cell transplant (HSCT) recipients. When it occurs, this fungal infection is a major limitation for a successful transplant. This review comprehensively analyses 24 cases, reported in the literature, of patients with haematological malignancies including leukemias, multiple myeloma, and lymphomas, as indication for HSCT, who presented with cryptococcosis after transplantation. Of the 24 cases, 11 each occurred in patients receiving allogeneic and autologous stem cell transplants, from bone marrow, peripheral blood, and umbilical cord blood. HSCT recipients were slightly more often male, and the age of the patients ranged from 12 to 74 years. Antifungal prophylaxis was reported in most cases. Clinical manifestations of cryptococcal disease included more frequently central nervous system involvement followed by fungaemia, disseminated infection, pulmonary cryptococcosis, cerebellitis, and diarrhea. Diagnosis differed depending on the clinical presentation but habitually included cryptococcal antigen assay, India ink, and culture. Notably, not only *C. neoformans* but also *C. albidus*, *C. terreus*, *C. laurentii*, and *C. adeliensis* were identified as the causal species, the last two including strains resistant to fluconazole. Amphotericin B, alone or in combination, was the most common antifungal drug used for the treatment of cryptococcosis in HSCT recipients. Due to the small number of cases, it was not possible to establish if mortality rate, which was the same as survival rate, depends on the effect of the immunosuppressive regimen, the site of cryptococcal infection, and/or the antifungal therapy used to control the mycosis. Although uncommon, the recognition of cryptococcal disease in stem cell transplant is essential for a timely and adequate treatment, improved prognosis, reduced morbidity and mortality, and successful transplantation.

## 1. Introduction

In transplant recipients, fungi have become important pathogens, mainly due to the patients' immunosuppression. An impaired immune function increases the risk of disseminated infections and worsens the outcome of the underlying disease, contributing substantially to morbidity and infection-related mortality after transplantation [1]. In addition, not only the antifungal agents used for prophylaxis and therapy are associated with toxicity and side effects but the use of these antifungal drugs concomitantly with immunosuppressant agents has adverse effects, mostly due to harmful interactions [2]. In most cases, fungal infections have a significant medical and economic burden that far exceeds that expected of the underlying disease alone.

*Cryptococcus neoformans*, an encapsulated yeast acquired from the environment, is one of the most common opportunistic fungus that can affect different organ systems in transplant patients. In solid organ transplant (SOT) recipients, cryptococcal disease is the third most frequent invasive fungal infection (IFI), usually because of the high doses of corticosteroids and other immunosuppressive agents that depress cellular immune responses in these patients [3, 4]. Additionally, hematopoietic stem cell transplant (HSCT) recipients have also a risk of infectious complications by *Cryptococcus*. Even though cryptococcosis in HSCT occurs much more rarely, this clinical presentation is relevant because if there is no suspicion to diagnose and treat cryptococcal infection, this can be life-threatening, especially in patients with central nervous system (CNS) involvement. Although in general, most cryptococcal infections are caused by *C. neoformans*, and several cryptococcal cases in HSCT recipients have been caused by non-*neoformans Cryptococcus* species [4, 5].

In contrast with the epidemiological data of cryptococcosis in SOT recipients, the epidemiology concerning HSCT recipients is not well characterized due to the relative rarity of cases, the cause of which remains uncertain [4, 5]. This review describes the distinctly unusual cases of cryptococcal disease in HSCT recipients and summarizes the clinical characteristics, methods and technology used for diagnosis and treatment. Although there are some analyses and data about the incidence of IFI in HSCT recipients, available in the Transplant Associated Infection Surveillance Network (TRANSNET) [6], the information about cryptococcosis in these groups of transplanted patients is rather limited. This is the most comprehensive review that brings together all published cases of cryptococcosis after stem cell transplantation, highlighting the recognition that this mycosis deserves, even though it occurs uncommonly among transplanted patients with haematological malignancies.

## 2. Clinical Cases

Currently, 24 cases of cryptococcosis in HSCT recipients have been reported in the English literature, with the first case reported in 1994 and the last in 2019 [7–27] (Table 1). Although not always documented, leukemias (acute myelogenous, acute lymphoblastic, and myelodysplasia) [8, 9, 13, 19, 20, 24, 27], multiple myeloma [11, 15, 17, 23], and lymphomas (Hodgkin disease and other lymphomas) [12, 18, 23, 25, 26] were the common haematological malignancies that were indications for HSCT, as reported worldwide [28]. From the patients with available data, 11 received allogeneic stem cell transplant, and 11 were autologous HSCT recipients. When reported, stem cells for both autologous and allogeneic transplantation were obtained from peripheral blood (*n* = 3), bone marrow (*n* = 3), and umbilical cord blood (*n* = 2), the last one including one patient who underwent a double cord HSCT (Table 1). Allogenic transplant recipients were more frequently male than female (5 vs. 3), while male patients were slightly less common than females in autologous transplantation (4 vs. 5). Overall, the median age of the patients was 47 with a range from 12 to 74 years (Table 2).

Before HSCT, treatment for the haematological malignancies included primarily chemotherapy and in some cases body irradiation [9, 12, 17, 24, 26]. As stated in 13 cases, chemotherapy agents were always used in combination. Although the drugs used, their dosage, the frequency, and duration of treatments differed among patients, most regimens included cyclophosphamide in combination or sequentially with other antineoplastic agents or with corticosteroids medications such as dexamethasone and prednisone. BeEAM regimen (carmustine, etoposide, cytarabine, and melphalan) was used as a conditioning protocol in a patient with Hodgkin's lymphoma [25]. Antithymocyte globulin, as induction therapy [13], and cyclosporine, as immunosuppressant medication, in combination with prednisone [19] were reported in two patients with acute myelogenous leukemia. Antifungal prophylaxis was reported in 12 cases. HSCT recipients received fluconazole more frequently (*n* = 6), including a patient that received both fluconazole and amphotericin B. Caspofungin prophylaxis was given to two patients, while ketoconazole, itraconazole, and micafungin were reported in one case each. Preemptive antifungal therapy without specification of the drug was indicated in two cases (Table 1).

Time to onset of cryptococcosis greatly differed among the transplanted patients. Cryptococcal infection was diagnosed from the day after admission for the stem cell transplant [13] until 5 years after the transplantation [19]. In addition, some differences were observed in the clinical presentation of cryptococcosis (Tables 1 and 2). Patients presented more frequently with CNS involvement (52.2%), fungaemia (17.4%), and disseminated disease (13%) than with pulmonary cryptococcosis (8.7%). One patient presented cryptococcal meningitis followed by fungaemia [26], and another patient had disseminated cryptococcosis with renal involvement [20]. One case of cryptococcal cerebellitis [15] and one case of diarrhea [25], which are unusual presentations of cryptococcosis, were also reported. Symptoms before fungal diagnosis were very broad, including from fever, headache, vomiting, confusion, loss of consciousness, and hypertension to more complex and severe symptoms such as limb paresthesia, erythematous nodular rash, skin lesions, seizures, and even thrombotic microangiopathy.

The outcome of the patients was registered in 20 cases. Interestingly, from those, 10 reported that the patient died and in 10 cases, the patient survived. Thus, overall mortality rate was the same as the survival rate. However, among the fatal cases, patients receiving allogenic transplantation were more common than patients with an autologous transplant (7 vs. 2). In one fatal case, the type of transplant was not stated. Inversely, among the survivors, patients with an allogenic transplant were less common than patients receiving autologous transplantation (3 vs. 7) (Table 1). Graft-versus-host disease (GvHD) was reported in three patients who died [9, 19, 20] and one patient who survived [26]. Among the 10 patients who recovered, two were reported with neurological sequelae [18, 23] and two were reported to be treated with fluconazole for an indefinite period of time [11, 15]. Apart from the differences in the outcome of patients according with the type of transplant received, due to the small number of cases of cryptococcosis in HSCT, it was not possible to establish if mortality rate depended on the effect of the immunosuppressive regimen, the site of cryptococcal infection, and/or the antifungal therapy used to control the mycosis. When specified, poor outcomes were associated with multiorgan [13] and respiratory failure [17, 19], a worsened neurological status that led to coma [9] and deteriorated conditions [20]. Cause-specific mortality, attributable to cryptococcosis, was not documented in any case.

## 3. Diagnosis

In HSCT recipients suspected of having CNS involvement, prompt lumbar puncture with measurement of cerebrospinal fluid (CSF) opening pressure and rapid cryptococcal antigen assay were performed, as this is the preferred diagnosis approach for cryptococcal meningitis [29]. Detection of polysaccharide antigen (CrAg) in CSF was performed either by latex agglutination (LA) or lateral flow assay (LFA). CSF India ink examination and/or CSF culture were also performed, although not all cases were positive. In one case, CSF was cultured in *Guizotia abyssinica* agar, a differential-selective medium [13]. Magnetic resonance imaging (MRI) [16, 18] and computed tomographic (CT) scan [9, 17] were also used to diagnose some cases. When antigen detection, India ink examination and CSF culture were negative, the diagnosis of cryptococcal meningitis was established by positive PCR for *Cryptococcus* and detection of cryptococcal antigen in serum [19].

In transplanted patients with nonmeningeal cryptococcal disease, such as fungaemia and disseminated cryptococcosis, blood culture was the most commonly used diagnostic method, sometimes combined with CrAg assay in serum and pleural fluid. In a patient with a mucocutaneous presentation of disseminated infection, biopsy of the tongue, the affected organ, suggested *Cryptococcus*, which was confirmed by tissue culture, positive CrAg in serum and CSF, and positive cultures from CSF and blood [17]. Pulmonary involvement was diagnosed by microscopy of bronchoalveolar lavage (BAL) to observe the yeast, and respiratory samples were also used for culture and CrAg detection [27]. In the case of cryptococcal-associated diarrhea, diagnosis was achieved by observation of large yeast cells in Gram staining and by culturing stool samples [25].

Species identification was performed when the yeast was recovered from CSF, blood, or other biological samples, by using automatized systems based on biochemical assimilation tests such as Vitek [8, 24, 25] and the ID 32C yeast identification system from bioMérieux (France) [13]. Molecular methods, such as PCR and sequencing of the internal transcribed spacer (ITS) region, the D1/D2 region, and the intergenic spacer (IGS) region of the ribosomal RNA gene of the isolated yeasts, were also utilized to determine the species [13, 19, 26].

In HSCT recipients with cryptococcosis, *C. neoformans* was the most common causal species (61.9%) [11, 15–21, 23, 26, 27], followed by *C. laurentii* (14.3%) [8, 24, 25], *Cryptococcus* sp. (9.5%) [14, 22], and one case each (4.8%) of *C. adeliensis* [13, 19], *C. albidus* [12], and *C. terreus*, the last species causing infection simultaneously with *Candida tropicalis* [7]. In three cases, the aetiological agent was not determined or not stated (Table 2). Antifungal susceptibility testing was performed only in four cases. In one report, both broth microdilution and the e-test were used to describe the strain of *C. adeliensis* as susceptible to amphotericin B, but resistant to flucytosine and fluconazole, and with reduced susceptibility to itraconazole and voriconazole, compared to the *C. neoformans* reference strains [13]. By using the Sensititre™ YeastOne™ panel, the minimum inhibitory concentration (MIC) for eight antifungal drugs was determined for the *C. laurentii* strain, which had a high MIC to fluconazole (8 *μ*g/ml) [25]. Although the other two cases did not report the method used for susceptibility testing, both reported *C. neoformans* strains susceptible to fluconazole [20, 26].

## 4. Treatment

Antifungal therapy for the management of cryptococcosis in HSCT recipients was reported in 18 out of the 24 cases. From those, the mainstay of treatment in 15 cases (83.3%), independently of the clinical presentation, was amphotericin B used as mono (*n* = 4) or polytherapy (*n* = 11). In combination, amphotericin B deoxycholate or the lipid-associated formulation, liposomal amphotericin B, was used with 5-flucytosine alone [9, 23, 26, 30] or together with fluconazole [11, 15, 18, 23]. One patient received amphotericin B plus itraconazole [12], and two patients received more than four antifungal drugs, including amphotericin B, 5-flucytosine, fluconazole, voriconazole, and posaconazole [16, 20]. Even though ketoconazole was used in one patient as preemptive therapy, once the diagnosis of cryptococcemia was performed, the antifungal treatment was changed for fluconazole alone [8]. Monotherapy with voriconazole was also reported in a patient receiving fluconazole as antifungal prophylaxis [25]. Antifungal treatment without specification of the drug was mentioned in one case [17] (Table 1).

## 5. Discussion

Cryptococcal disease is very rare following stem cell transplantation. In the literature, only 24 cases of cryptococcosis in HSCT recipients have been reported in a period of 26 years. Data from TRANSNET revealed that in HSCT patients, the incidence of this mycosis was only 0.6% in a period of six years, which was comparable to the incidence of endemic fungi such as *Histoplasma*, *Blastomyces*, and *Coccidioides* [6]. This number is markedly low compared with the almost 250,000 cases of cryptococcal meningitis that are estimated to occur globally every year among adults living with HIV [31]. However, cryptococcosis in HIV-negative individuals, including HSCT recipients, is usually characterized for delayed diagnosis, poor prognosis, and long-term neurologic sequelae [32], which represents a major hindrance to the success of the transplantation. In addition, when a fungal infection is acquired, HSCT recipients will require not only chemotherapy with or without total-body irradiation but also antifungal treatment to achieve clinical remission.

Despite common environmental exposure to *C. neoformans* and other cryptococcal species, disease is rare in the healthy population because of high natural resistance. While the first line of defence against the yeasts is the innate immune system, T cell-mediated immunity is the predominant component of the host defence mechanism against the fungus. Thus, cryptococcosis is a well-described infection in patients with diseases causing T cell deficiency, such as AIDS or lymphomas [33]. The rarity of this mycosis in HSCT recipients, especially compared with that of SOT recipients, might be explained since during thymic regeneration of transplanted stem cells, there is proliferation of Th1 cells, which results protective against cryptococcosis. In addition, widespread use of antifungal prophylaxis in the early posttransplant period may account for less frequent occurrence of cryptococcal disease in this group of transplanted patients [5, 34].

In a review that analyzed nine cases of cryptococcal infection after stem cell transplantation, a higher risk to acquire this mycosis was attributable to autologous than to allogenic transplants, for unknown reasons [5]. Conversely, the current review, which includes more than double the amount of cases, shows that there were no differences regarding the number of allogeneic and autologous transplant recipients. In fact, the small number of cases in either review does not allow establishing which type of transplant has higher or lower risk for developing cryptococcosis. The higher number of fatal cases among patients receiving allogenic transplantation compared to those with an autologous transplant, however, could be attributed to several factors. In allogenic recipients, immune reconstitution is much slower, and there is a greater risk of life-threatening complications as they have risk of GvHD, mismatched transplants, and the need for additional immunosuppressive therapy to prevent GvHD and graft rejection [35, 36]. Regarding the gender of the HSCT recipients and contrary to different reports that have consistently described a predominance of males among individuals with cryptococcosis, including organ transplant recipients [10], this review shows that the amount of males and females receiving stem cell transplantation did not differ considerably (9 vs. 8, respectively). As cryptococcosis is rare among HSCT recipients, it is not possible to determine the basis for the roughly equal prevalence of this presentation among male and female patients. Yet, the slightly higher frequency of male patients among allogenic recipients could be explained considering that from patients submitted to allogenic HSCT, around 60% are male recipients, as shown in large-scale cohorts studies [35, 37, 38].

In general, antifungal prophylaxis in patients undergoing HSCT depends on the timing of transplantation and on the prevalence of the infectious complications. During the preengraftment period, *Candida* species infections predominate, which are favoured by mucositis induced by immunosuppressive drugs. However, during the same period, chemotherapy-induced neutropenia predisposes patients to other fungal infections, especially by *Aspergillus* species. In the early and late postengraftment periods, the alteration of cellular immunity caused by GvHD and its associated treatments, together with the inadequate immune reconstitution, which can last up to 12 months, represent major risks for mycosis caused by *Pneumocystis jirovecii* and moulds such as *Aspergillus* spp., *Fusarium* spp., and Mucormycetes (formerly Zygomycetes) [34]. Thus, the clinical guidelines recommend the use of fluconazole or micafungin as first-line preemptive therapy in neutropenic HSCT recipients. As alternative therapies, voriconazole, posaconazole, or lipid presentations of amphotericin B are recommended among allogenic transplantation, and in patients with significant GvHD, posaconazole is the prophylactic antifungal of choice, with voriconazole, echinocandins, and lipid presentations of amphotericin B, as alternatives [39]. Even though cryptococcosis in patients receiving stem cell transplants is uncommon, the general recommendations for antifungal prophylaxis in these patients are likely to be effective, except in the case of the use of echinocandins, as cryptococcal species are intrinsically resistant to this type of antifungal drugs [40].

As it is widely known, meningoencephalitis is the most common clinical manifestation of cryptococcosis [41]. When reported, in HSCT recipients, cryptococcal disease involved mainly the CNS, with more than half of the cases manifesting meningitis alone or, in one case, followed by fungaemia, which reflects the disseminated nature of the infection. As occurring in SOT recipients, the most common site of extraneural infection was the blood [4], with four cases having fungaemia at the time of presentation and three presenting with disseminated disease involving the tongue and the skin. Cryptococcosis limited to the lungs was less frequent, and among the plethora of clinical manifestations of cryptococcosis, cerebellitis and diarrhea were also reported. Concerning the outcome of the patients and due to the small number of cases, it was not possible to correlate the clinical presentations and onset of cryptococcosis, the causal cryptococcal species, and/or the antifungal prophylaxis or treatment, with an increased mortality rate.

Early diagnosis of cryptococcosis in all clinical presentations is of paramount importance, as this leads to a timely treatment, improved prognosis, reduced mortality, and in HSCT recipients particularly, to a successful transplantation. In stem cell transplant patients with cryptococcal meningitis, the diagnosis was achieved mainly by CrAg detection, which is one of the most successful immunological tests used to diagnose this mycosis [29]. However, although both LA or LFA are very sensitive and specific, these tests are not as sensitive when it comes to infections caused by non-*neoformans* species, very likely due to structural differences in the capsular polysaccharide antigens [42, 43]. Therefore, a negative CrAg test does not exclude infection by non-*neoformans* species, which in HSCT recipients accounts for almost 30% of cases. The India ink test in CSF was also used to diagnose cryptococcal meningitis in HSCT recipients; however, while this technique is simple and readily accessible in resource-limited settings, unfortunately, it also has a low sensitivity [44]. Independently of the clinical presentation, culture remains the gold standard for diagnosis, although it can take 3 to 7 days for the yeast to grow, which makes the final diagnosis slow, and sometimes low fungal loads can cause false negatives [44]. In HSCT recipients, only three cases reported the use of MRI and CT to help diagnose meningitis. Although CT and MRI could appear normal among patients with cryptococcal meningoencephalitis, 47% and 8% of cases, respectively, imaging could be useful to determine abnormal findings such as hydrocephalus, dilated Virchow Robin spaces, pseudocysts, and cryptococcosis, which are correlated with higher mortality [45]. Therefore, diagnostic imaging techniques should be performed in all patients with suspected or proven cryptococcosis.

While *C. neoformans* is the most common cause of cryptococcal disease in patients with immunosuppressive conditions, including transplant recipients, non-*neoformans* species are becoming increasingly important as etiologic agents of infections in immunocompromised patients, affecting the CNS, lungs, skin, and even causing fungaemia [42, 46]. Notably, *C. adeliensis*, *C. albidus*, *C. laurentii*, and *C. terreus* were reported as the cause of meningitis, disseminated cryptococcosis, and diarrhea in six HSCT recipients (Table 1). In most cases, identification was done by using automated biochemical systems, which are rapid and cover a wide range of clinically important microorganisms. In one case, birdseed agar, which allows differentiating the brown/black colonies from *C. neoformans* compared to white/creamy colonies from other non-*neoformans* species, was used together with ITS sequencing, for a most accurate species identification.

Treatment of cryptococcosis in patients undergoing HSCT has been extrapolated from the management of this disease in patients with SOT. Thereby, the initial treatment of meningitis, disseminated forms, and moderate-to-severe pulmonary cryptococcosis is recommended to be lipid amphotericin B combined with 5-flucytosine. Fluconazole is indicated as consolidation therapy and for mild-to-moderate forms of pulmonary cryptococcosis [47]. However, in HSCT recipients with cryptococcal disease, amphotericin B was reported as monotherapy in four cases, three of which were fatal. In addition, amphotericin B deoxycholate, which has been associated with nephrotoxicity [48], was also used. Interestingly, voriconazole was successfully used as monotherapy to treat diarrhea caused by *C. laurentii*, since the strain had a high MIC to fluconazole and as the patient was already on fluconazole prophylaxis [25]. This highlights the importance of carrying out antifungal susceptibility testing and the significance of incorporating this testing into clinical practice. For some yeasts, including *Cryptococcus* spp., fluconazole resistance has been associated with therapeutic failure [49]. Considering that fluconazole is recommended to be used alone for consolidation therapy, it is used as primary prophylaxis, and it has even been used as a single drug for the treatment of cryptococcal meningitis, depending on drug availability [29], it is remarkable to identify strains with reduced susceptibility or resistance to this azole.

## 6. Conclusions

Fungal infections continue to be major threats to patients undergoing HSCT as they are associated with high fatality rates. Although rare, cryptococcosis is part of these mycoses, usually characterized by late diagnosis, poor prognosis, long-term neurologic sequelae, and higher doses of antifungal drugs, which hampers the success of transplantation. Therefore, early recognition is essential for prompt treatment, complete recovery, and better outcomes. The identification of infrequent cryptococcal species causing disease, together with antifungal susceptibility testing, is also of paramount importance, mainly because of the emergence of strains and species that are resistant to commonly used antifungal drugs. Further research, delving into the possible causes of this uncommon but important mycosis in stem cell recipients, is therefore necessary.

## Figures and Tables

**Table 1:** Reported cases of hematopoietic stem cell transplant recipients with cryptococcosis.

Transplant source	Age/gender	Underlying condition	Antifungal prophylaxis	Time to onset	Clinical presentation	Species	Treatment	Outcome	Year [ref.]
-/BM	—	—	FLC	—	Cryptococcemia	*C. terreus * ^*∗*^	AMB	Dead	1994 [7]
Autologous/BM	17/M	Leukemia	KTC	1 month	Cryptococcemia	*C. laurentii*	FLC	Alive	1997 [8]
Allogeneic/BM	12/F	ALL	None	64 days	Meningitis	—	AMBd; 5-FC	Dead	1997 [9]
Autologous	—	—	—	—	Pulmonary involvement	—	—	—	2001 [10]
Autologous	—	—	—	—	—	—	—	—	2001 [10]
Autologous	42/F	MM	FLC	4 months	Meningitis	*C. neoformans*	L-AMB; 5-FC; FLC	Alive	2002 [11]
Autologous/PB	51/M	PTCL and AML	FLC, AMB	0 days	Cryptococcemia	*C. albidus*	AMB; ITC	Alive	2003 [12]
Allogeneic/PB	40/F	AML	ITC	8 days	Meningitis	*C. adeliensis*	L-AMB; 5-FC	Dead	2004 [13]
—	—	—	CAS	12 days	Cryptococcemia	*Cryptococcus* sp.	—	—	2007 [14]
Autologous	61/F	MM	—	2 years	Cryptococcal cerebellitis	*C. neoformans*	AMB; 5-FC; FLC	Alive	2009 [15]
Allogeneic	18/M	—	—	3 years	Meningitis	*C. neoformans*	L-AMB; FLC; VRC; PSC; 5-FC	Alive	2009 [16]
Autologous	65/M	MM	Unspecified	7 months	Disseminated cryptococcosis	*C. neoformans*	Unspecified	Dead	2012 [17]
Autologous/PB	41/M	PTCL	FLC	9 days	Meningitis	*C. neoformans*	L-AMB; 5-FC; FLC	Alive	2014 [18]
Allogeneic	64/M	AML	—	5 years	Meningitis	*C. neoformans*	AMB	Dead	2016 [19]
Allogeneic/DC	31/M	ALL	CAS	1 year	Disseminated cryptococcosis with renal involvement	*C. neoformans*	L-AMB; 5-FC; FLC; VRC	Dead	2016 [20]
Allogeneic	—	—	—	50 days	Meningitis	*C. neoformans*	—	Dead	2016 [21]
Allogeneic	—	—	—	538 days	Meningitis	*C. neoformans*	—	Dead	2016 [21]
Allogeneic	—	—	Unspecified	-	Meningitis	*Cryptococcus* sp.	—	—	2016 [22]
Autologous	74/F	DLBCL	—	22 days	Meningitis	*C. neoformans*	AMB; 5-FC; FLC	Alive	2017 [23]
Autologous	69/F	MM	—	893 days	Meningitis	*C. neoformans*	AMB; 5-FC	Dead	2017 [23]
Allogeneic	47/F	AML	FLC	3 years	Disseminated cryptococcosis	*C. laurentii*	AMBd	Alive	2017 [24]
Autologous	26/F	HL	FLC	4 days	Diarrhea	*C. laurentii*	VRC	Alive	2017 [25]
Allogeneic/UC	55/M	FL	MFC	51 days	Meningitis with fungemia	*C. neoformans*	L-AMB; 5-FC	Alive	2018 [26]
Allogeneic	66/M	MDS	—	18 days	Pulmonary involvement	*C. neoformans*	L-AMB	Dead	2019 [27]

BM, bone marrow; PB, peripheral blood; DC, double cord; UC, umbilical cord; ALL, acute lymphoblastic leukemia; AML, acute myelogenous leukemia; DLBCL, diffuse large B-cell lymphoma; FL, follicular lymphoma; HL, Hodgkin's lymphoma; MDS, myelodysplastic syndrome; MM, multiple myeloma; PTCL, peripheral T-cell lymphoma; AMBd, amphotericin B deoxycholate; CAS, caspofungin; FLC, fluconazole; ITC, itraconazole, KTC, ketoconazole; L-AMB, liposomal amphotericin B; MFC, micafungin; PSC, posaconazole; VRC, voriconazole; 5-FC, 5-flucytosine. ^*∗*^Simultaneously with *Candida tropicalis*.

**Table 2:** Demographic and clinical characteristics of hematopoietic stem cell transplant recipients with cryptococcosis and characteristics of the disease.

Characteristic	Number (%)
Gender (*n* = 17)	
Male	9 (52.9)
Female	8 (47.1)
Primary hematologic disease (*n* = 16)	
Multiple myeloma	4 (25.0)
Acute myelogenous leukemia	3 (18.8)
Acute lymphoblastic leukemia	2 (12.5)
Peripheral T-cell lymphoma	2 (12.5)
Diffuse large B-cell lymphoma	1 (6.3)
Follicular lymphoma	1 (6.3)
Hodgkin's lymphoma	1 (6.3)
Myelodysplastic syndrome	1 (6.3)
Unspecified leukemia	1 (6.3)
Transplant type (*n* = 22)	
Allogeneic	11 (50.0)
Autologous	11 (50.0)
Site of infection (*n* = 23)	
CNS	12 (52.2)
Cryptococcemia	4 (17.4)
Disseminated	3 (13.0)
Pulmonary	2 (8.7)
Other	2 (8.7)
Outcome (*n* = 20)	
Dead	10 (50.0)
Alive	10 (50.0)
Species (*n* = 21)	
*Cryptococcus neoformans*	13 (61.9)
*Cryptococcus laurentii*	3 (14.3)
*Cryptococcus* sp.	2 (9.5)
*Cryptococcus adeliensis*	1 (4.8)
*Cryptococcus albidus*	1 (4.8)
*Cryptococcus terreus*	1 (4.8)
	Median (range)
Age (years) (*n* = 17)	47 (12–74)
Time of onset after HSCT (days) (*n* = 20)	57.5 (0–1825)

*n*, number of cases with available data from the patients.

## Data Availability

The data supporting this review are from previously reported studies and datasets, which have been cited. The processed data are available from the corresponding author upon request.
